# The Path to Therapeutic Furin Inhibitors: From Yeast Pheromones to SARS-CoV-2

**DOI:** 10.3390/ijms23073435

**Published:** 2022-03-22

**Authors:** Gary Thomas, Frédéric Couture, Anna Kwiatkowska

**Affiliations:** 1 Department of Microbiology and Molecular Genetics, University of Pittsburgh School of Medicine, Pittsburgh, PA 15219, USA; 2 TransBIOTech, Lévis, QC G6V 6Z3, Canada; frederic.couture@tbt.qc.ca; 3 Institute of Nutrition and Functional Foods, Laval University, Quebec, QC G1V 0A6, Canada; 4 Centre de Recherche du Centre Intégré de Santé et de Services Sociaux de Chaudière-Appalaches, Lévis, QC G6V 3Z1, Canada; 5 Institut de Pharmacologie de Sherbrooke, Université de Sherbrooke, Sherbrooke, QC J1H 5N4, Canada

**Keywords:** furin, proprotein convertase, insulin, cancer, atherosclerosis, anthrax, HIV-1, SARS-CoV-2, protease inhibitor, α_1_-PDX

## Abstract

The spurious acquisition and optimization of a furin cleavage site in the SARS-CoV-2 spike protein is associated with increased viral transmission and disease, and has generated intense interest in the development and application of therapeutic furin inhibitors to thwart the COVID-19 pandemic. This review summarizes the seminal studies that informed current efforts to inhibit furin. These include the convergent efforts of endocrinologists, virologists, and yeast geneticists that, together, culminated in the discovery of furin. We describe the pioneering biochemical studies which led to the first furin inhibitors that were able to block the disease pathways which are broadly critical for pathogen virulence, tumor invasiveness, and atherosclerosis. We then summarize how these studies subsequently informed current strategies leading to the development of small-molecule furin inhibitors as potential therapies to combat SARS-CoV-2 and other diseases that rely on furin for their pathogenicity and progression.

## 1. Introduction

When I penned the first comprehensive review on furin 20 years ago [1], the USA was reeling from the deliberate dissemination of *Bacillus anthracis* spores through the mail, and Asia was in turmoil from the emergence of the highly pathogenic H5N1 avian influenza virus which was capable of infecting humans. Caught ill-prepared, the USA resorted to dispatching swarms of biohazard containment teams to cordon off contaminated areas, whereas the Hong Kong government was forced to extinguish all the poultry in the region to prevent further zoonotic transfer. Perplexed by these calamitous events, I rhetorically asked “what next?” [1]. The answer: SARS-CoV-2. Exploiting a devious strategy long used by other pathogenic viruses, SARS-CoV-2 acquired a consensus furin site in its spike proprotein, greatly increasing viral transmission [2]. The recognition of furin as a virulence factor for disparate microbial pathogens ranging from *Bacillus anthracis* to SARS-CoV-2, and as a player in diseases ranging from atherosclerosis to cancer spurred the development of furin inhibitors as a novel broad-based therapeutic strategy. The goal of this short review is to summarize the key discoveries that led to the identification of furin as the prototypic member of the proprotein convertase (PC) family—a nearly 70-year endeavor—and, in turn, the generation of the first furin inhibitors capable of attenuating furin-dependent disease pathways. These pioneering studies laid the foundation for the generation of additional design strategies leading to the development of peptidomimetic and small-molecule furin inhibitors.

## 2. A Diabetic Dog, Sterile Yeast, and a Pox Virus Led to the Identification of Furin

As with many avenues in biomedical research, the path to experimental furin inhibitors began in 1921 with the Nobel-prize-winning work of Fred Banting, a medical instructor at the University of Western Ontario (now Western University), and Charles Best, an undergraduate assistant in John Macleod’s lab at the University of Toronto, who together conducted the seminal experiment that led to the discovery of insulin [3,4]. Working under John Macleod’s supervision, they perfected a two-step pancreatectomy method that allowed them to rescue glucose homeostasis by injecting a diabetic dog with an islet extract isolated from a healthy donor dog. Aided by the expertise of James Collip, a biochemist from the University of Alberta, the team soon purified insulin from islets and demonstrated its effectiveness in treating type I diabetics. The large-scale production of insulin isolated from feedlot animals by Ely Lily revolutionized the treatment of diabetes [5].

During the 1940s, Fred Sanger at Cambridge University perfected methods necessary to sequence proteins [6,7]. The ready supply of insulin produced by the pharmaceutical industry provided Sanger with the ideal protein to test this methodology. In a series of studies that led to Sanger’s first Nobel Prize, his team reported the primary structure of insulin and discovered that it is composed of two peptide chains, that is, a 30-amino acid B chain and a 21-amino acid A chain, covalently linked by a pair of interchain disulfide bridges [8,9]. The importance of these studies cannot be overstated, as Sanger not only reported the first protein sequence, but also suggested that each protein would have its own arrangement of amino acids, thus marking the birth of molecular biology [5].

**Figure 1:**
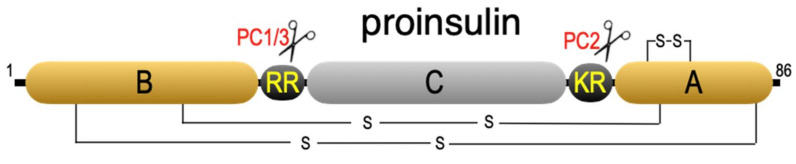
Proinsulin. Shown are the disulfide bonds and the sites cleaved by PC3 (PC1/3) and PC2.

The question of how the two disulfide-linked insulin chains could assemble with such efficacy was puzzling. Attempts to join the two chains with the correct disulfide pairs failed miserably [10]. In 1967, Don Steiner at the University of Chicago solved this dilemma. Supplied with patient-derived β-cell tumors, Steiner conducted a pulse-chase study which demonstrated that insulin is synthesized as an approximately 10 kDa prohormone, proinsulin, which is subsequently converted to the disulfide-linked two-chain insulin hormone (Figure 1) [11]. Sequencing the proinsulin molecule revealed that the N-terminal B chain and C-terminal A chain sequences were joined by a connecting peptide (C-peptide) and flanked by doublets of basic amino acids (-ArgArg^32^- at the B/C junction and -LysArg^65^- at the C/A junction) [12,13].

**Figure 2:**
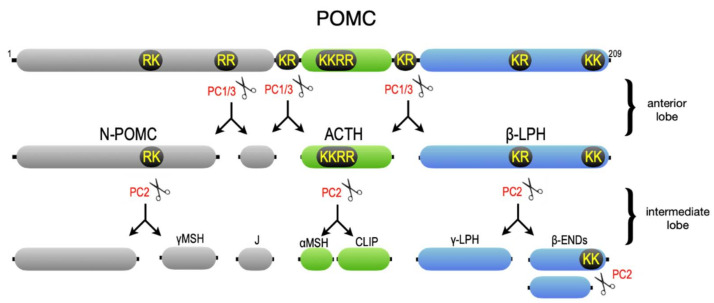
POMC. Shown are the processing steps that occur in the pituitary anterior lobe and neurointermediate lobe. The sites cut by proprotein convertases PC1/3 and PC2 are shown. For a recent review, see [20].

Intensive biochemical studies coupled with advances in cDNA cloning soon revealed that virtually all peptide hormones and neuropeptides are synthesized as prohormone molecules, many of which harbor sequences for multiple bioactive peptides, which are linked together by doublets or clusters of basic amino acids and are proteolytically excised in a tissue-specific manner [14,15]. One notable example is a study by Ed Herbert and his lab at the University of Oregon (and later at the Vollum Institute) that described the tissue-specific processing of POMC (proopiomelanocortin) in the pituitary gland (Figure 2) [16]. In the anterior lobe of the pituitary, POMC is processed at doublets of basic amino acids to generate ACTH (adrenocorticotropin), which stimulates cortisol release from the adrenal gland, and β-LPH (β-lipotropin), which was suggested to have lipolytic activity [17]. In the intermediate lobe, however, ACTH is cleaved at a cluster of basic amino acids to yield α-MSH (α-melanocyte-stimulating hormone), which modulates functions ranging from feeding to pigmentation. In addition, β-LPH is further cleaved at a doublet of basic amino acids to generate γ-LPH and β-endorphin, one of the principal opioid peptides. To ascertain the endoprotease “signatures” of these endocrine cells, Barbara Thorne in my lab analyzed a battery of POMC cleavage site mutants expressed in primary endocrine cells [18,19]. She suggested that just two endoproteases were broadly responsible for most prohormone processing steps in endocrine and neuroendocrine cells, including the activation of relatively simple prohormones such as insulin in the pancreas, as well as the tissue-specific processing of complex prohormones such as POMC in the pituitary.

Understanding how diverse proproteins are activated required the identification of the proteases that catalyze these reactions. Capitalizing on methodologies developed by Sanger, Don Steiner found that exposure of proinsulin to mild trypsin digestion excised the mature insulin hormone. This led to the suggestion that proinsulin is processed in the mildly acidic late secretory pathway compartments of the cell by the sequential activities of a trypsin-like endoprotease, frequently followed by trimming of the residual basic amino acids by a carboxypeptidase B-like activity to generate the mature peptide [21]. For the next quarter-century, numerous researchers sought to isolate the relevant endoproteases by conventional biochemical methods. A visit to the local abattoir and days in the cold room fractionating tissues yielded an overwhelming number of candidate activities, each capable of cutting protein or peptide substrates at basic amino acids under mildly acidic conditions. Unfortunately, these studies were lacking in a genetic approach to conclusively identify the bona fide converting endoproteases. As John Hutton (then at Cambridge University) lamented, “many candidates have been put forward—and eventually shot down” [22].

**Figure 3:**
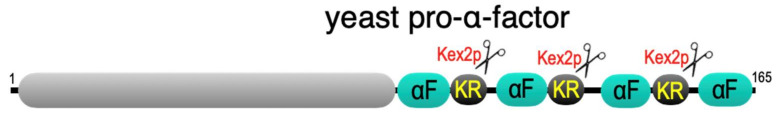
Yeast pro-α-mating factor. Shown are the four cryptic α-factor (αF) peptides and the flanking -LysArg- cleavage sites, which are processed by Kexp2.

The first breakthrough using a genetic approach was made by Ira Herskowitz’s lab, then at the University of Oregon, who found that, similar to many mammalian neuropeptide precursors, the yeast mating pheromone, α-factor, is encoded by a tandem of four copies of the tridecapeptide encased in a larger prohormone, pro-α-mating factor [23]. Each α-factor sequence is flanked on one side by a linker segment containing a -LysArg- doublet of basic amino acids, suggesting that yeast may express a prohormone processing endoprotease (Figure 3). Jeremy Thorner’s lab at UC Berkeley capitalized on these findings and demonstrated that yeast *Kex2* encodes a protease required for cleavage of the -LysArg- sites in pro-α-mating factor and pro-killer toxin [24]. They also raised the possibility that the Kex2 protein (Kex2p) may be the prototype for the mammalian prohormone convertases.

Leveraging advances in viral vector technology, we showed that, when expressed in mammalian cells, yeast Kex2p can correctly process POMC to sets of peptides generated in the pituitary gland (including γ-LPH and β-endorphin), demonstrating that Kex2p is functionally similar, and thus likely structurally similar, to the long-sought-after mammalian proprotein convertases [25,26]. A database search conducted by Robert Fuller, now at the University of Michigan, identified the first such human Kex2p homologue, an obscure open reading frame coined “furin” [27]. The Van de Ven team had serendipitously discovered the furin locus in 1986, designated *fur* (fes/fps upstream region), which they predicted would encode a membrane receptor [28]. In a series of papers beginning in 1990, Sean Molloy and Pat Bresnahan in my lab reported that furin is a *trans*-Golgi network (TGN) membrane-localized, calcium-dependent serine endoprotease that cuts the neurotrophin precursor pro-β-NGF at the multibasic site -Arg-X-Lys/Arg-Arg^↓^, which is a decisive step modulating neuron survival [29,30,31,32]. The number of proproteins identified as being cleaved by furin now exceeds 150 [33,34]. These range from TGF-β family members essential for embryogenesis to receptors, cell adhesion proteins, serum proteins, and other proteases involved in disease pathways ranging from cancer to cardiovascular disease, as well as a number of microbial proproteins critical for pathogen virulence [1,33,35,36,37,38].

The identification of furin as the first human proprotein convertase guided PCR strategies to identify additional members of the PC family [39]. As predicted by our earlier work [18,19], Laurel Thomas and Barbara Thorne, in collaboration with Don Steiner’s lab, demonstrated that PC2 and PC3 (also called PC1) are a common core of neuroendocrine proprotein convertases that cleave POMC to the complex sets of peptides found in the pituitary as well as proinsulin to insulin [40,41] (for a detailed description of the proprotein convertase family see [1,42]).

## 3. Furin Essentials

Furin’s broad role in proprotein activation is attributed to changes in its gene expression, as well as its highly regulated intracellular trafficking itinerary. For example, elevated furin expression negatively correlates with disease outcome in several cancers, increasing metastasis while reducing immune-cell infiltration [1,37,43,44]. Bioinformatic analyses identified *FURIN* polymorphisms as risk factors for diabetes, cardiovascular disease, obesity, and all-cause mortality [45,46,47]. Importantly, these furin-associated risk factors, together with furin’s key role in SARS-CoV-2 pathogenesis (as described below), may underpin the increased vulnerability of susceptible patients (including obese and diabetic patients) to negative outcomes from COVID-19 [48,49].

In cells, furin localizes to the TGN and traffics between this processing compartment and two other processing compartments: the cell surface and early endosomes [1]. In the TGN/biosynthetic pathway, furin activates many substrates, including TGF-βs, receptors, and viral envelope glycoproteins. Cell-surface furin activates cellular proteins involved in cell migration and tumor metastasis as well as several pathogen proteins, including anthrax protective antigen (PA), proaerolysin, and *Clostridium septicum* α-toxin [1,50,51]. The mildly acidic pH of early endosomal compartments enables profurin to complete its own autoproteolytic activation steps in a compartment-specific manner and is exploited by pathogens to activate A/B-type bacterial toxins, including *Pseudomonas* exotoxin A and Shiga toxin [1,52,53]. The trafficking of furin between these various processing compartments is mediated by sequences in its cytoplasmic domain, which bind several clathrin adaptors (i.e., AP-1, AP-2, and AP-4) and contain a pair of serine residues that are reversibly phosphorylated by the actions of protein kinase CK2 and specific isoforms of protein phosphatase 2A (PP2A) [1,54,55]. Phosphorylated furin binds the sorting protein PACS-1, which localizes furin to either the TGN or to a peripheral cycling loop between early endosomes and the cell surface [56,57]. Movement from early endosomes to the TGN requires the PP2A-dependent dephosphorylation of furin [55]. Furin molecules localized to the cell surface are tethered by the actin-binding protein filamin-A (ABP-280) [58]. Interestingly, in cancer cells hypoxia induces both furin expression and its redistribution to the plasma membrane, where it tethers to filamin-A and activates MT1-MMP/MM2P-dependent cell invasion [50,51].

Systematic analyses of the furin-dependent cleavage of two bona fide furin substrates (i.e., anthrax PA and virulent H5N8 influenza virus HA proteins), each containing defined amino acid changes surrounding their cleavage sites, identified -Arg-X-X-Arg-^↓^ as the minimal site required for efficient processing by furin [30,59,60,61]. Subsequent crystallography and enzymology studies reinforced these findings, identifying the residues in the catalytic domain that bind the critical Arg residues and demonstrating that favorable amino acids at the P2 and P6 subsites can compensate for less-favorable ones at P4 [62,63].

## 4. Pathogenic Viruses from Bird Flu to SARS-CoV-2 Acquire Furin Cleavage Sites to Increase Tropism

Pioneering studies in the 1980s by Robert Webster at St. Jude Research and Hans-Dieter Klenk at the University of Marburg converged on furin as a key factor regulating viral tropism [64]. They determined that the pathogenicity of avian influenza viruses correlated with the cleavability of its fusion protein precursor HA_0_ to generate the fusion-competent HA_1_–HA_2_ complex. Avirulent avian influenza viruses, which lack a consensus furin site in HA_0_, cause a localized infection in the intestinal tract. Mutation of the HA_0_ cleavage site to a consensus furin site enables the virus to be activated by ubiquitously expressed furin, allowing the virus to spread systemically throughout the bird [65]. Analysis of the H5N1 influenza virus, which caused the deadly flu outbreak in Hong Kong in 1997, revealed that just two mutations were required to generate the lethal virus, including the generation of a tandem furin site in the cleavage junction between HA_1_ and HA_2_ (-ArgGluArgArgArgLysLysArg-^↓^), which increases cleavability [66]. Increases in viral tropism resulting from the acquisition of a furin site have been reported for other pathogenic viruses [1,36].

**Figure 4:**
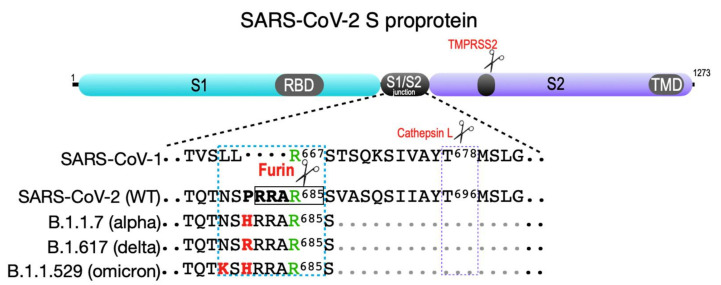
SARS-CoV-2 spike protein. Shown are the S1 and S2 segments in the SARS family S proprotein that flank the S1/S2 cleavage site junction as well as the ACE2 receptor-binding domain (RBD) in S1 and the transmembrane domain (TMD) in S2. The S2′ TMPRSS2 cleavage site is common to all SARS coronaviruses (violet box). The SARS-CoV-1 S1/S2 junction is cut by cathepsin L at Thr^678^ (see [74]). SARS-CoV-2 contains a four-amino-acid insertion (PRAA^684^), which converts the trypsin-sensitive Arg^685^ residue (green) to the P1 site cut by furin (RRAR^685^, boxed). The cyan box also shows the B.1.1.7 (alpha variant) furin site containing the P^681^ → H change; the more transmissible B.1.617.2 (delta variant) furin site, which contains the P^681^ → R change; and the recently reported B.1.1.529 (omicron variant), which contains both P^681^ → H and N^679^ → K changes.

SARS-CoV-2 illustrates in real-time how acquiring and then optimizing the furin cleavage site can have devastating consequences on morbidity and mortality. Both SARS-CoV and SARS-CoV-2 rely on the cleavage of their spike (S) proproteins by the cell surface protease TMPRSS2 to expose the receptor-binding domain (RBD) that contacts the primary virus receptor, ACE2 (Figure 4). However, TMPRSS2 cleavage does not fully expose the SARS-CoV-2 RBD [67]. To overcome this block, SARS-CoV-2 acquired a suboptimal furin site (-TNSP^681^RRAR^↓^S^686^-(furin cleavage site is underlined)) at the SARS-CoV-2 S1/S2 junction, which directs furin-dependent cleavage of the S proprotein to fully unmask the RBD, allowing it to efficiently bind ACE2 [67]. In addition, the exposed basic amino acids at the cleaved S1 C-terminus are not removed by a carboxypeptidase but instead bind the co-receptor neuropilin-1, further augmenting viral transmission [68,69]. The B.1.1.7 (alpha) variant contains a P^681^ → H change (-TNRH^681^RRAR^↓^S^686^-), which increases cleavability by furin [70,71]. This strategically placed His residue likely becomes protonated following the transit of the S protein to late secretory pathway compartments, providing a positive charge that triggers cleavage by furin in endosomal compartments [53]. The highly transmissible B.1.617.2 (delta) variant contains a P^681^ → R change (-TNSR^681^RRAR^↓^S^686^-), creating a permanent positive charge at residue 681 and further increasing furin-dependent infectivity, possibly in multiple cellular compartments [70,71]. Intriguingly, the recently described B.1.1.529 (omicron) variant appears to have “doubled-down” on the endosomal processing by furin through the addition of both the P^681^ → H and N^679^ → K changes (-TK^679^RH^681^RRAR^↓^S^686^-). This double change reinstates reliance on the pH-sensitive His^681^ as initially observed with B.1.1.7, but likely further increases cleavage efficiency in endosomes through the addition of the positive charge at Lys^679^—a nefarious mimicry of the efficacious compartment-specific autoactivation pathway employed by furin [53]. These possibilities await experimental testing, and may illuminate the endomembrane itinerary used by SARS-CoV-2 to optimize or limit virus assembly and transmissibility. Intuitively, one would predict that an Ala^684^ → Arg change (-TNSPRRR**^684^**R^↓^S^686^-) would maximize transmission efficiency. While this change increases syncytia formation in vitro, it nonetheless impedes virus entry [72]. Similar findings were reported for HIV-1 gp160, which also maintains an unfavorable acidic amino acid at the P3 site (-ArgGluLysArg-^↓^) [73]. Thus, maintenance of a suboptimal furin site may best support subsequent conformational changes in an envelope-protein-specific manner for optimal infectivity.

## 5. The First-Generation Furin Inhibitors

The identification of furin as the principal cellular endoprotease that cleaves viral envelope glycoproteins prompted the development of furin inhibitors able to block viral pathogenicity. These initial studies relied on two markedly different strategies [75,76]. Elliot Shaw at the Friedrich Miescher Institute, together with Hans-Dieter Klenk, developed an irreversible peptidyl chloromethyl ketone (CMK) active site furin inhibitor. Their strategy was based on Shaw’s work from the 1960s which demonstrated that attaching the substrate P1 amino acid to the CMK warhead produced inhibitors selective for different classes of serine proteases by reacting with the active site histidine: Tos-Phe-CH_2_Cl (TPCK) inhibits chymotrypsin and Tos-Lys-CH_2_Cl (TLCK) inhibits trypsin [77,78,79]. Incorporating furin’s consensus cleavage sequence, Shaw generated furin-directed CMKs (e.g., dec-Arg-Glu-Lys-Arg-CH_2_Cl) [76]. A decanoyl group was added to increase membrane permeability. Treatment of cells with dec- Arg-Glu-Lys-Arg -CMK blocked the processing of HIV-1 gp160 and the production of infectious HIV-1 as well as several other pathogenic viruses, including paramyxovirus, pathogenic avian influenza viruses, and SARS-CoV-2 [60,76,80,81]. Unfortunately, the furin-directed CMKs lack specificity and are low nanomolar inhibitors of all proprotein convertases, reducing their utility [82].

In an alternative strategy, my lab engineered a variant of the serpin α_1_-antitrypsin that is highly selective for furin called α_1_-antitrypsin Portland (α_1_-PDX) [75]. Circulating α_1_-antitrypsin (α_1_-AT) is the primary inhibitor of neutrophil elastase, protecting the lungs during the acute-phase response to tissue injury. Mechanistically, α_1_-AT functions as a suicide substrate [83]. This globular serpin contains a metastable 19-amino acid reactive center loop (RCL) that corresponds to the P15-P4′ residues spanning the target enzyme cleavage site. The formation of the protease—α_1_-AT acyl intermediate invokes a massive conformational rearrangement of the RCL, which traps the bound protease in a stable complex. The RCL can be engineered to have high target specificity, as demonstrated by a unique case in the 1970s, where a boy from Pittsburgh was diagnosed with a fatal posttraumatic bleeding disorder caused by a Met^358^ → Arg mutation at the P1 site in the α_1_-AT RCL (Ala-Ile-Pro-Met^358^ → Ala-Ile-Pro-**Arg^358^**) [84,85]. This single amino acid change in this α_1_-AT mutant, called α_1_-antitrypsin Pittsburgh (α_1_-PIT), had two profound effects. It switched the serpin from an elastase inhibitor to a potent thrombin inhibitor, explaining the fatal acute-phase bleeding disorder and highlighting the target specificity that can be accommodated by the RCL. In addition, α_1_-PIT was associated with circulating proalbumin, which requires cleavage at an -ArgArg- site, leading the authors to speculate that α_1_-PIT encounters and inhibits the proalbumin convertase during transport through the hepatocyte secretory pathway [86].

Eric Anderson and Francois Jean, now at the University of British Columbia, determined that α_1_-PIT was a weak inhibitor of furin [75,82]. However, changing the RCL to the minimal Arg-X-X-Arg consensus furin site (Ala-Ile-Pro-**Arg^358^** → **Arg**-Ile-Pro-**Arg^358^**) generated an engineered serpin, α_1_-PDX, which is a highly selective subnanomolar inhibitor of furin, which no longer recognizes elastase or thrombin. A_1_-PDX can be expressed from the nucleus, or it can be generated in bacteria and either applied to cells in vitro or systemically delivered to mice. Using these delivery approaches, α_1_-PDX blocks the furin-dependent activation of several pathogenic viruses, including HIV-1, measles virus, and HCMV, as well as the furin-dependent activation of *Pseudomonas* exotoxin A [82,87,88]. α_1_-PDX is effective in cancer models, inhibiting tumor cell invasiveness in vitro and tumor metastasis in vivo [43,89,90,91]. Finally, systemic administration of the recombinant α_1_-PDX reduced atherosclerotic progression in vivo, in part by inhibiting the furin-dependent activation of MT1-MMP/MMP2 [92]. The primary obstacle to the development of α_1_-PDX, or any form of α_1_-AT, as a potential therapeutic has been the lack of an expression system that can generate the physiologically stable α_1_-AT protein. However, new advances in formulating α_1_-AT and its variants have overcome this challenge [93]. Together with recent work suggesting that donor-purified α_1_-AT inhibits the TMPRSS2-dependent cell entry of SARS-CoV-2 [94], these advancements raise the exciting possibility that combinations of recombinant α_1_-AT and α_1_-PDX may be a potential treatment for SARS-CoV-2 [93,95].

## 6. The Promise of Small-Molecule Furin Inhibitors as Broad-Based Therapeutics

The successes achieved by using peptidyl CMKs or α_1_-PDX to inhibit furin have guided additional approaches toward the development of therapeutic furin inhibitors [96]. Leveraging Elliott Shaw’s success with peptidyl CMKs and the furin crystal structure, several cell-penetrant peptide inhibitors, including substrate-based inhibitors containing the H5N1 cleavage site and poly-D-arginine-based peptides, have been developed and shown to prevent the processing of anthrax PA in cellulo and to protect mice from anthrax toxemia in vivo [97,98,99,100,101]. In addition, structure–activity relationship (SAR) analyses have been performed to improve molecular stability and cell penetrance, yielding various peptidomimetic inhibitors containing a decarboxylated P1 Arg, a replacement of the P2 and P4 Arg by canavanine, an addition of P5 Arg mimetics, or the addition of azaβ^3^ moieties to the N- and C-termini, and leading to nanomolar furin inhibitors that prevent pathogen activation [102,103,104,105,106,107]. Importantly, the discovery of small-molecule furin inhibitors, notably a series of 2,5-dideoxystreptamine derivatives with nanomolar potency that protect cells from anthrax PA toxicity, represents a critical next step in the realization of therapeutic furin inhibitors [108,109,110]. A recent study suggesting the bioavailable small-molecule furin inhibitor BOS-981 blocks cleavage of the SARS-CoV-2 S1/S2 site and reduces viral titer further supports this approach [111]. The next few years should reveal which of these approaches may lead us closer to the realization of a broad-based therapeutic that selectively targets furin to combat multiple diseases.

## Data Availability

Not applicable.
